# Continuous transanal decompression for infants with long- and total-type Hirschsprung’s diseases as a bridge to curative surgery: a single-center experience

**DOI:** 10.1186/s40792-017-0318-y

**Published:** 2017-03-10

**Authors:** Kyoko Mochizuki, Masato Shinkai, Norihiko Kitagawa, Hiroshi Take, Hidehito Usui, Takashi Hosokawa, Kaori Yamoto

**Affiliations:** Department of Surgery, Kanagawa Children’s Medical Center, 2-138-4 Mutsukawa, Minami-ku, Yokohama, 232-0066 Japan

**Keywords:** Transanal decompression, Long-segment type, Total type, Hirschsprung’s disease, Children

## Abstract

**Background:**

The purpose of this study is to assess the usefulness of continuous bowel decompression using an indwelling transanal tube (ITT) for preoperative management in infants with long-segment (L)- or total (T)-type Hirschsprung’s disease (HD).

**Case presentation:**

Between 2012 and 2015, seven patients with L- or T-type HD underwent preoperative bowel management by continuous bowel decompression using an ITT during waiting period for curative surgery. Continuous bowel decompression was done using an ITT, a 10–12F flexible dual lumen tube placed through the rectum up to the dilated colon under fluoroscopic guidance and secured to the bilateral buttocks. The ITT tips were located at least in a dilated colon or the cecum if there was no radiographic transitional zone. The ITT was left open for continuous drainage, and its patency was checked by regular suction until the curative operation. The patient status and complications of this preoperative management were reviewed retrospectively.

**Results:**

The median duration of decompression management was 65 (17–137) days. During decompression period, neither abdominal distention, enterocolitis, nor other complications occurred and six patients could stay at home until the curative operation. The weight-for-age *Z*-score at curative surgery was the same as or higher than that at birth in five patients. ITT replacement was needed three times per patient on an average for accidental ITT removal, ITT stenosis, or ITT hardening.

**Conclusions:**

Bowel management by continuous bowel decompression using an ITT is easy, safe, and effective for preoperative management in patients with L- or T-type HD and may permit single-stage surgery rendering colostomy or enterostomy unnecessary.

## Background

Hirschsprung’s disease (HD) is a congenital bowel motility disorder that occurs in approximately one in every 5000 live births. Although short-segment (S)-type HD is diagnosed earlier and the incidence of preoperative enterocolitis has decreased [1, 2], the diagnosis of long-segment (L)- and total (T)-type HD is frequently delayed [2, 3]. Contrast enema appears to be most accurate in the cases of S-type HD (usually defined as having a transitional zone at or distal to the rectosigmoid junction); however, contrast enema is not always useful for diagnosing and detecting a transitional zone in patients with L- and T-type HD [2, 3]. The transitional zone in L-type HD is difficult to identify by radiography, and the colon in T-type HD might even look normal [4]. Even though early diagnosis of L- and T-type HD might be difficult, delay of management for those types of HD should be avoided to prevent enterocolitis.

In infants with S-type HD, preoperative colonic preparation is usually manageable using a glycerin enema, serial rectal decompression, or washouts, whereas most infants with longer type aganglionosis require diverting stoma (colostomy or enterostomy) followed by staged surgeries to complete curative treatment. These staged operations are physical and psychological burdens on the patients in terms of more than a single general anesthesia, laparotomy, and long hospital stays [5, 6]. Also, stoma management may be associated with risk of stoma complications and financial burden of ostomy aids.

**Fig. 1:**
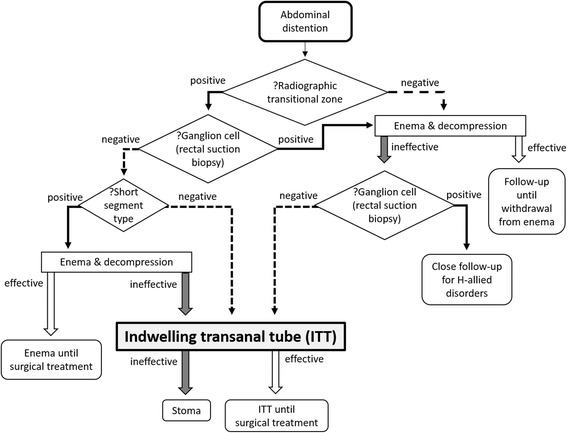
Diagnosis and management for Hirschsprung’s disease in our hospital. In our hospital, patients were diagnosed with HD and preoperatively managed according to the decision tree

In our hospital, patients diagnosed with HD are preoperatively managed according to the decision tree (Fig. 1). Since 2012, we have adopted preoperative management by continuous colonic decompression using an indwelling transanal tube (ITT) to avoid staged operations as well as management delay for infants with L- and T-type HD. Our method using the ITT usually involves continuous drainage, regular suction to check its patency, and irrigation of the tube if necessary. Here, we review our experience of preoperative management using an ITT.

Additionally, we have chosen curative surgical procedure and its timing depending on the site of a pathological transitional zone, which is confirmed intraoperatively. Soave procedure was performed for those with the transitional zone distal to the mid-transverse colon when they weigh above 3 kg, and Duhamel procedure was chosen in patients with the transitional zone proximal to the mid-transverse colon when they weigh above 6 kg and the automatic suturing device is applicable.

## Case presentation

Between January 2012 and December 2015, 23 patients with HD were admitted and managed in our hospital. Two patients had emergency stoma because of sepsis or perforation at first visit. Nineteen patients had caliber change on contrast enema and were diagnosed with HD by suction biopsy. Preoperatively, 13 of them could be managed by glycerin enema and serial rectal decompression, and pathologically diagnosed with S-type HD at curative surgery. The remaining six patients were unresponsive to glycerin enema and serial rectal decompression, and the other two patients without apparent caliber changes on contrast enema were effectively managed using ITT before curative surgery. All the eight patients gave informed consent for the procedures. Among these eight patients, seven patients were diagnosed with L- or T-type HD and one patient was diagnosed with S-type HD at curative surgery. In this study, the L type was a HD with the transitional zone at or proximal to the descending colon and the T type was the one with the transitional zone at or proximal to the ileum end.

**Fig. 2:**
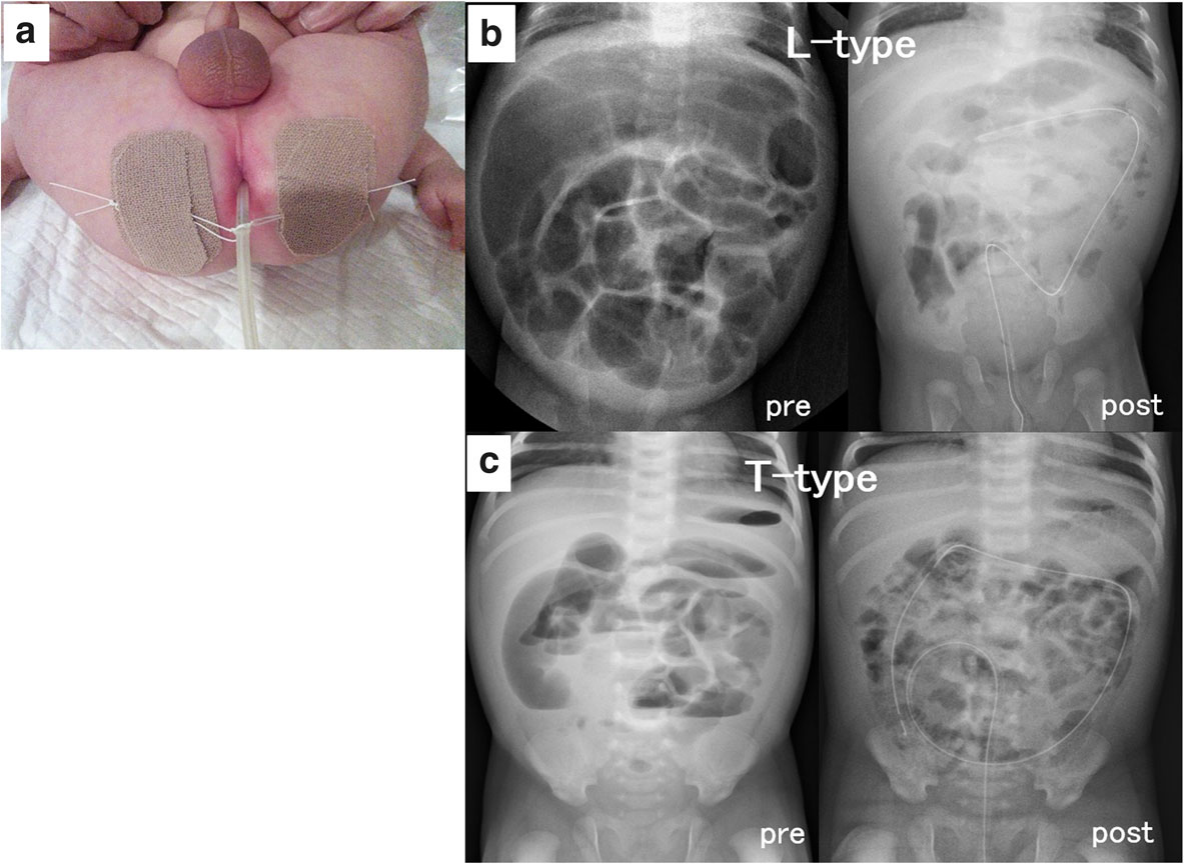
Indwelling transanal tube (ITT). **a** A 10–12F flexible drainage tube was placed and secured to the bilateral buttocks. **b** Decompression using an ITT in long-segment (L)-type Hirschsprung’s disease; abdominal X-ray taken before decompression showing a dilated colon (*pre*), which disappeared after ITT insertion (*post*). **c** Decompression using an ITT in total (T)-type HD; abdominal distention is visibly improved after (*post*) versus before (*pre*) ITT insertion

Decompression by an ITT was done by placing a 10–12F Salem Sump™ Dual Lumen tube (Covidien, Dublin) through the rectum into the dilated colon under fluoroscopic guidance and securing it to the bilateral buttocks with tape (Fig. 2a). The ITT tips were located at least in a dilated colon or in the cecum if there was no caliber change in the colon (Fig. 2b, c). The ITT was left open for continuous drainage wrapped in a diaper and suctioned manually at every diaper changing time to check its patency until curative operations.

The status and complications of the seven patients with L- or T-type HD who underwent continuous bowel decompression using an ITT before curative surgery were assessed.

### Results

Table 1 shows the patient characteristics of the seven cases. The management period was distributed from April 2012 to November 2015. The median birth weight and gestational age was 2894 g (1466–4092 g) and 38 weeks (31–40 weeks), respectively. There were six cases of L-type HD and one case of T-type HD. Regarding congenital anomalies, there were three patients with trisomy 21 and two patients with congenital heart diseases. Transitional zones were located at the descending colon in four patients, transverse colon in two patients, and ileum end in the other patient. The median body weight at curative surgery was 4674 g (3300–7348 g), and laparoscopic-assisted Soave procedure was selected in six patients and laparoscopic-assisted Duhamel procedure was selected in the other patient as curative surgical procedure.

**Table 1:** The characteristics in patients

Case	BW (g)	GA (w)	Congenital anomaly	Type	Transitional zone	Curative surgery	
						BW (g)	Surgical procedure
1	2894	40	Trisomy 21 VSD	Long	D colon	3300	LA Soave
2	3184	38	–	Long	D colon	3502	LA Soave
3	1466	31	Trisomy 21	Long	T colon	4288	LA Soave
4	3036	39	–	Long	D colon	7100	LA Soave
5	4092	39	–	Total	Ileum end	6978	LA Duhamel
6	2290	38	Trisomy 21	Long	D colon	4674	LA Soave
7	2252	37	PDA	Long	T colon	7348	LA Soave
Median	2894	38				4674	

*BW* birth weight, *GA* gestational age, *VSD* ventricular septal defect, *PDA* patent ductus arteriosus, *D colon* descending colon, *T colon* transverse colon, *LA* laparoscopic assisted

**Fig. 3:**
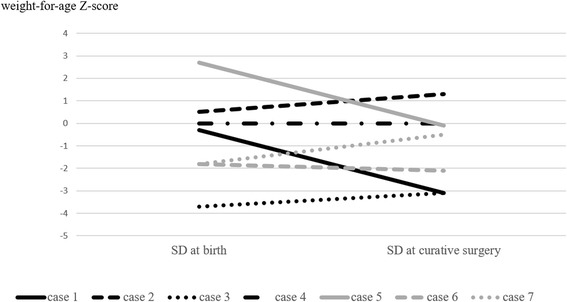
Body weight-for-age growth chart. The weight-for-age *Z*-score at curative surgery became the same as or higher than that at birth in five patients

The median ages at the start of decompression, curative surgery, and duration of decompression management were 13 days (6–84 days), 128 days (24–173 days), and 65 days (17–137 days) (Table 2). The weight-for-age *Z*-score at curative surgery was the same as or higher than that at birth in five patients (Fig. 3).

**Table 2:** Management using an ITT

Case	Age at start of decompression (day)	Age at curative surgery (day)	Duration of decompression management (day)	Management at home	ITT replacement (times)	ITT trouble (times)
1	24	71	47	Yes	2	Removal (2)
2	7	24	17	No	1	Stenosis (1)
3	84	128	44	Yes	1	Removal (1)
4	13	138	125	Yes	4	Removal (3) Hardening (1)
5	10	143	133	Yes	4	Removal (4)
6	35	100	65	Yes	6	Removal (4) Hardening (1) Stenosis (1)
7	6	173	167	Yes	3	Hardening (2) Stenosis (1)
Median	13	128	65		3

The ITT tip sites were the T colon in four cases and the cecum in the other three cases. ITT replacement was needed three times per patient on average because of accidental removal of ITT, stenosis of ITT, or hardening of ITT (Table 2). During decompression, no cases of abdominal distention, enterocolitis, or any other complications excluding ITT troubles occurred and six patients kept staying at home and visited the outpatient clinic every month until the curative operation. One patient could not stay home because of parental refusal. Even in a T-type HD case, a continuous bowel decompression management using an ITT was effective without any complications excluding ITT troubles.

Although additional suction or irrigation was to be carried out in case of intractable abdominal distention, none of those measures were needed during ITT management in any patient.

The perianal skin was exposed to small amount of stool passing through the anus around the ITT during continuous bowel decompression; however, perianal dermatitis was preventable by washing and barrier ointment. Perianal dermatitis after curative pull-through surgery, such as skin erosion and redness, was noticed in only one or two cases.

### Discussion

Regarding preoperative management for HD, Hirose et al. [5] and Kato et al. [7] reported the cases of two babies and 16 babies with rectal to transverse colonic aganglionosis who underwent preoperative decompression and irrigation via an ITT while waiting for primary pull-through procedures. In Hirose’s decompression method [5], a balloon catheter was inserted to transverse colon and the catheter was connected to a pinch-cock and lavage bottles for continuous drainage and colonic irrigation with 100 ml warm saline was done twice a day. The catheters were replaced about every 10 days. In Kato’s decompression method [7], the same tube as ours was inserted to the rectum, sigmoid colon, or transverse colon and the tube was left open and wrapped in a diaper and colonic irrigation with 300 ml warm saline was done twice a day. The tubes were replaced about every 14 days. Their mean preoperative tubing time was 48 and 54 days, respectively, and these preoperative managements may have been performed during hospitalization. There were no episodes of enterocolitis in both reports.

Our study supported their experience and confirmed the effectiveness of preoperative bowel decompression by ITT for longer aganglionosis-type HD and for longer duration with quite infrequent irrigation required. Moreover, our management was feasible at home. In our cases, ITT replacement was needed four times in three patients because of hardening of ITT. We replaced the tube not regularly within several weeks but when the tube was found hard at once-a-month outpatient clinic. Moreover, radiation exposure was decreased by avoiding regular replacement of the tube.

Using ITT management, the patients avoided at least two operative procedures and hospitalization (for construction and closure of colostomy) and financial burden of ostomy goods. Moreover, some patients who needed heart surgeries may have evaded intra- and postoperative contamination possibly caused by stomas.

Continuous bowel decompression management using an ITT was simple, less invasive, and lower cost compared with diverting stoma. Moreover, this management did not disturb the patients’ growth as shown by their body weight gain during the waiting period for curative surgery. In the case of malposition or wear-out failure of an ITT, some tubes had to be repositioned or replaced during the waiting period, which was easily carried out at our outpatient clinic.

## Conclusions

Bowel management by continuous bowel decompression using an ITT is easy, safe, and effective for preoperative management in patients with L- or T-type HD and may permit single-stage surgery rendering colostomy or enterostomy unnecessary.

## Abbreviations

BW: Birth weight
GA: Gestational age
HD: Hirschsprung’s disease
ITT: Indwelling transanal tube
L: Long segment
LA: Laparoscopic assisted
PDA: Patent ductus arteriosus
S: Short segment
T: Total
VSD: Ventricular septal defect
